# Subluxation: dogma or science?

**DOI:** 10.1186/1746-1340-13-17

**Published:** 2005-08-10

**Authors:** Joseph C Keating, Keith H Charlton, Jaroslaw P Grod, Stephen M Perle, David Sikorski, James F Winterstein

**Affiliations:** 1 6135 North Central Avenue, Phoenix, AZ, 85012, USA; 2 School of Medicine, Mayne Medical School, University of Queensland, Herston, Queensland 4006, Australia; 3 Department of Graduate Education and Research, Canadian Memorial Chiropractic College, 6100 Leslie Street, Toronto ON, M2H 3J1, Canada; 4 Department of Clinical Sciences, College of Chiropractic, University of Bridgeport, 225 Myrtle Ave., Bridgeport, CT 06604, USA; 5 Department of Chiropractic Procedures, Southern California University of Health Sciences, 16200 E. Amber Valley Drive, Whittier, CA 90604, USA; 6 President, National University of Health Sciences, 200 East Roosevelt Road, Lombard, IL 60148, USA

## Abstract

Subluxation syndrome is a legitimate, potentially testable, theoretical construct for which there is little experimental evidence. Acceptable as hypothesis, the widespread assertion of the clinical meaningfulness of this notion brings ridicule from the scientific and health care communities and confusion within the chiropractic profession. We believe that an evidence-orientation among chiropractors requires that we distinguish between subluxation dogma vs. subluxation as the potential focus of clinical research. We lament efforts to generate unity within the profession through consensus statements concerning subluxation dogma, and believe that cultural authority will continue to elude us so long as we assert dogma as though it were validated clinical theory.

## Background

### Status of a Construct

More than twenty years ago Donald K. Moon, D.C. wrote of a "flight from the subluxation" among chiropractors [1]. Dr. Moon, a firm believer in the validity of the traditional chiropractic lesion, bemoaned the dearth of scientific data to substantiate the construct, and warned of the possibility that medical researchers would step in to fill the void created by chiropractors' indolence. He decried the tendency among many chiropractors to pit diagnosis against spinal analysis (i.e., subluxation-detection), as though the two were mutually exclusive.

In the years since, some members of the profession have developed scientific skills, and a literature bearing on the usefulness of spinal manipulation, generated by chiropractors and others, has evolved [e.g., [2-5]]. In the United States several chiropractic colleges have been the recipients of federal funds for scientific investigations, and a consortial center for investigations has been established at Palmer College of Chiropractic with federal money. University-based chiropractic schools have been established in several nations [6], and the scholarly works of chiropractors are now much more widely disseminated in chiropractic and non-chiropractic periodicals. The profession may look upon these developments and say with some pride that, indeed, there is a small but meaningful scientific literature in chiropractic [7,8].

Despite these accomplishments, many chiropractors preeminent theoretical construct remains unsubstantiated [9-11], and largely untested [12]. This lack of evidence may reflect a lack of interest among those with research skills; Nelson [11] observed that "clinical studies of the effectiveness of spinal manipulation are conducted and reported without reference to the presence or absence or even the existence of subluxations". The chiropractic subluxation stands pretty much today as it did at the dawn of the 20th century: an interesting notion without validation. And, as it has throughout the past century, D.D. Palmer's mediating variable remains a "bone of contention" between many chiropractors and the scientific community, as well as among chiropractors themselves.

Although books and monographs have been written about the presumed entity [e.g., [13-16]], and intra-professional political consensuses [17-19] have been reached on fuzzy conceptual definitions and unjustified claims (Table 1), little if any substantive experimental evidence for any operational definition of the chiropractic lesion has been offered in clinical trials. Notwithstanding strong intra-professional commitment to the subluxation construct [20,21] and reimbursement strategies that are legally based upon subluxation [22], there is today no scientific "gold standard" (10) for detecting these reputedly ubiquitous and supposedly significant clinical entities, and inadequate basic science data to illuminate the phenomenon [11,23]. The chiropractic subluxation continues to have as much or more political than scientific meaning [24].

**Table 1:** Assertions about subluxation offered by several chiropractic organizations [17-19]

Association of Chiropractic Colleges 4.0 The Subluxation Chiropractic is concerned with the preservation and restoration of health, and focuses particular attention on the subluxation. A subluxation is a complex of functional and/or structural and/or pathological articular changes that compromise neural integrity and may influence organ system function and general health. A subluxation is evaluated, diagnosed, and managed through the use of chiropractic procedures based on the best available rational and empirical evidence.		
		
Chiropractic Association of Australia ...We recognise and respect a universal intelligence (or order) in all matter and an innate intelligence within a living organism that strives to preserve life and, if uninhibited, will express optimal well being. We recognise that the practice of chiropractic focuses on the relationship between structure (primarily the spine) and function (as coordinated by the nervous system) and how that relationship affects the preservation and restoration of health. We recognise that subluxations compromise the expression of innate intelligence, and that prevention and removal of subluxations will facilitate the expression of optimal health. We respect, care about and are committed to the individual's holistic well being and emphasise the inherent recuperative power of the body to heal itself without the use of drugs or surgery. We respect and value the importance of intellectual honesty, scientific and academic excellence and the maintenance of integrity in serving the individual, the community and the profession.		
		
New Zealand Chiropractors' Association Chiropractors use a technique of correcting vertebral subluxations called an adjustment. An adjustment is a carefully executed manoeuvre that usually results in a joint clicking as a sticky joint is released. Adjustments are usually painless, and enjoyable, because the improved mobility is usually immediately noticeable, and the health benefits are noticed soon after.		
Benefits of Chiropractic Care		
Feel Great Relief from Pain Improves Immunity Restores Nerve Supply More Energy Restores Mobility	Improves Athletic Performance More efficient Body Function Allows Better Sleep Back to Work Faster Improves Posture No Drugs	Slows the Aging Process No Surgery Quicker Recovery No Needles Add Life to Years Add Years to Life
How It Works Chiropractic is based on the scientific fact that your nervous system controls the function of virtually every cell, tissue, organ and system of your body. While the brain is protected by the skull, the spinal cord is more vulnerable, covered by 24 moving vertebrae. When these bones lose their normal motion or position, they can irritate the nervous system. This disrupts the function of the tissues or organs these nerves control, and this is called vertebral subluxation complex. Chiropractic is the science of locating these areas of spinal malfunction and the art of correcting them to allow the body to heal itself. As we all know, regardless of which type of doctor you consult, only the body can heal itself.		

**Figure 1:**
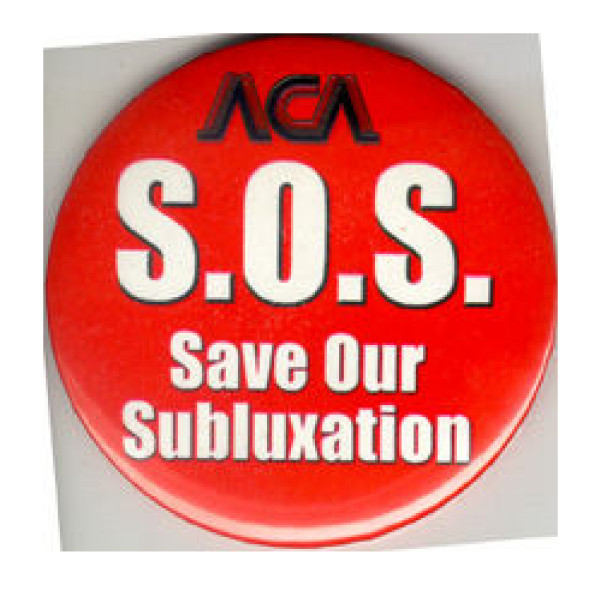
Political statement rendered on a button by the American Chiropractic Association, 2003.

We believe that Dr. Moon's concerns were only partly justified. All in all, there has been no flight from the subluxation on the part of the field or its leaders [e.g., [25,26]], nor much move towards it either (on the part of the profession's scholars) [12]. The profession – its rank-and-file and political leadership (see Figure 1) – has not abandoned the subluxation as an *a priori *principle guiding many of its activities. The chiropractic subluxation and subluxation-related beliefs permeate the practice of chiropractic, the marketing rhetoric offered by many chiropractors, the legal and political strategies pursued by various trade associations, and the sense of identity for many in the profession... all this for a hypothetical construct whose relevance for health and illness has yet to be established.

The traditional chiropractic lesion has not been the focus of systematic clinical research for the purpose of determining its meaningfulness (or lack thereof). In the absence of scientific validation, the propagation of unsubstantiated claims for many chiropractors favorite mediating variable is an obstacle to scientific credibility and cultural authority for the profession. It is our purpose to remind the profession of the implications and consequences of offering subluxation dogmatically, rather than as a plausible and testable proposition.

## Discussion

### The Dogma of Subluxation

*The spinal subluxation, though we have been correcting it with spinal adjustment for 100 years, is not fully understood. Scientific research presently is not sophisticated enough to determine the neurophysiological impact that spinal subluxation has on our patients. Does that mean that we do not adjust our patients because it has not been proven? Absolutely not. I treat my patients as if each spinal adjustment has a virtually unlimited potential in improving their health... *[27].

So wrote a member of the American Chiropractic Association's (ACA's) governing board in the centennial year of the profession. We might applaud the good doctor for acknowledging the inadequacy of basic research bearing on the subluxation; on the other hand, no recognition is given that the clinical meaningfulness of subluxation has yet to be established. One can only speculate what it means to treat every patient "as if each spinal adjustment has a virtually unlimited potential."

The dogmatic character of subluxation beliefs is exemplified by several assertions offered by the Association of Chiropractic Colleges (ACC) (see Table 1). Intended as a means of fostering greater unity among the chiropractic colleges, the ACC's "Paradigm" statement on subluxations has since been widely endorsed by national and international membership societies [28]. In effect, the ACC Paradigm has become the standard (if not official) position of a broad segment of the profession. There are several problems with the Paradigm.

First, the hypothesis that subluxation is some "complex of functional and/or structural and/or pathological articular changes that compromise neural integrity" is offered without qualification, that is, without mention of the tentative, largely untested quality of this claim. (As well, a stubbed toe would seem to meet the fuzzy criteria provided by the ACC.) The nature of the supposed compromise of "neural integrity" is unmentioned.

Secondly, the dogmatism of the ACC's unsubstantiated claim that subluxations "may influence organ system function and general health" is not spared by the qualifier "may." The phrase could mean that subluxations influence "organ system function and general health" in some but not all cases, or that subluxation may *not *have any health consequences. Although the latter interpretation is tantamount to acknowledging the hypothetical status of subluxation's putative effects, this meaning seems unlikely in light of the ACC's statement that chiropractic addresses the "preservation and restoration of health" through its focus on subluxation. Both interpretations beg the scientific questions: do subluxation and its correction "influence organ system function and general health"?

Lastly, the ACC claims that chiropractors use the "best available rational and empirical evidence" to detect and correct subluxations. This strikes us as pseudoscience, since the ACC does not offer any evidence for the assertions they make, and since the sum of all the evidence that we are aware of does not permit a conclusion about the clinical meaningfulness of subluxation. To the best of our knowledge, the available literature does not point to any preferred method of subluxation detection and correction, nor to any clinically practical method of quantifying compromised "neural integrity," nor to any health benefit likely to result from subluxation correction.

All in all, the ambiguities that permeate the ACC's statements on subluxation render it inadequate as a guide to clinical research. Although Wenban [29] proposes that the ACC statements on subluxation might be construed as "a very simplified map, for starting to find the future practice-relevant research priorities for chiropractic," he offers no suggestion that ACC's "map" is any improvement upon existing proposals for subluxation research strategies [e.g., [10,23,30]]. Owens [31] suggests that consensus models of subluxation are "useless for research purposes." Concerning the ACC's statements about subluxation, a signatory to the document asserts, "This paradigm was never intended to be a testable research hypothesis. It was constructed by a process of consensus to serve as a collective political statement, not a research hypothesis" [32]. More to the point of research need is a validated operational definition of subluxation [31]. Nelson [33] advises that "Whether chiropractors are actually treating lesions, or not, is a question of immense clinical and professional consequence. Resolution of the controversy will not be found through consensus panels nor through semantic tinkering, but through proposing and testing relevant hypotheses."

Whether the ACC's subluxation claims have succeeded as a political statement is beyond our concern here. These assertions were published as a priori truths (what many chiropractors have traditionally referred to as "principle"), and are exemplary of scientifically unjustified assertions made in many corners of the profession [34-36]. It matters not whether unsubstantiated assertions are offered for clinical, political, scientific, educational, marketing or other purposes; when offered without acknowledgment of their tentative character, they amount to dogmatism.

We contend that attempts to foster unity (among the schools or in the wider profession) at the expense of scientific integrity is ultimately self-defeating. To be sure, the profession's lack of cultural authority is based in part upon our characteristic disunity. However, attempts to generate unity by adoption of a common dogma can only bring scorn and continued alienation from the wider health care community and the public we all serve.

### Subluxation Semantics

The subluxation is identified by a great many names [37], but neither the abundance of labels nor efforts to reach consensus on terminology tell us anything about the validity of the construct. Nelson [11] points out that "...framing the subluxation debate as a semantic issue, resolvable by consensus, is precisely the same as asking whether we should refer to the spaceships used by aliens as flying saucers or UFOs." Neither adoption nor rejection of the term subluxation or any of its myriad synonyms will resolve the problem created by assuming a priori that subluxation is clinically meaningful. If and when we demonstrate that there are alien spaceships hovering over us, we suspect an appropriate terminology will develop on its own.

The clinical meaningfulness, if any, of subluxation cannot be established by definition. The notion that subluxation is inherently pathological, perhaps because some dictionary equates subluxation with ligamentous sprain, does not mean that joint dysfunction merits clinical intervention. Skin tags too might be considered pathological, but the mere presence of aberration or abnormality does not indicate a serious or treatment-worthy health problem. (The unfortunate lesson of decades of surgical intervention for bulging discs, performed in the hope of relieving back pain, seems all too frequently lost on many chiropractors.) We cannot establish the clinical meaningfulness of subluxation merely by branding it pathological; such would be word magic.

This is not to say that efforts to develop a standardized lexicon among chiropractors [e.g., [38]] are without merit. We think it important and useful, for example, to distinguish between the "orthopedic subluxation" [39] vs. "subluxation syndrome" [38]. The former is a more or less observable phenomenon recognized within and beyond chiropractic's borders. The latter is a theoretical notion, which relates subluxation of joints to deleterious health consequences, and is a testable, but largely untested proposition. This is no small distinction.

### Subluxation in Practice

As a pragmatic matter, subluxation refers to the target of many chiropractors manual interventions, and the individual practitioner may select from a range of theories, techniques and supposed clinical implications of the traditional chiropractic lesion. The latter include subluxation as a cause of musculoskeletal problems, as an etiological factor in various internal disorders and behavioral/psychological problems, and as a strategic intervention site for disease prevention and wellness enhancement. Hundreds of brand-name techniques have been offered for the purpose of correcting subluxations [13], but the clinical usefulness of subluxation correction has yet to be experimentally demonstrated.

The diversity of altered function attributed to subluxation and "nerve interference" parallels in some respects the "nervism" [40] and "spinal irritation" [41] of nineteenth century neurology and physiology. When coupled with vitalistic concepts of "Innate Intelligence," subluxation theories expand upon the "nature-trusting heresy" [42] of those earlier times. Unlike the therapeutic nihilism recommended by some nineteenth century physicians, many chiropractors' faith in nature gives rise to extensive regimens of subluxation correction [43]. The breadth of contemporary, uncritical speculations bearing on subluxation is captured in the boast of a chiropractic leader: "Rigor mortis is the only thing we can't help" [44]. Seaman [45] argues that "many chiropractic practices are guided by dogmatism instead of philosophy and science." In short, many chiropractors practice as though subluxation is clinically relevant, but seemingly without recognition that maybe it's not. When challenged, many chiropractors respond not with data, but by avowing "the chiropractic principle": subluxation.

The National Board of Chiropractic Examiners offers that: "By manually manipulating vertebrae into their normal physiological relationship, chiropractic practitioners relieve interference with the nervous system along with accompanying symptoms. This correction of joint dysfunction reestablishes normal mobility and comfort... Chiropractors see patients with spinal subluxations and joint dysfunction on a daily basis..." [[46], pp. 2, 53]. Chiropractors list "spinal subluxation/joint dysfunction" as the most frequent of all "conditions" they encounter [[46], pp. 53, 84, 101].

The magic and mystery of subluxation theories all too frequently direct the chiropractor's attention away from the legitimate question of whether subluxation (or any other rationale for manipulation) may be relevant in a patient's health problem, to a search for the "right" vertebra. Individual clinicians derive subluxation theories about particular spinal regions as "keys" to better health or to the resolution of particular disorders. For example, the subluxation sites for which adjustment has been suggested to relieve enuresis range from heads to tails [47-56]. Disciples of B.J. Palmer often restrict themselves to the upper cervical spine, while adherents to Logan's Basic Technique tend to focus on the sacrum. Sacro-occipital technique practitioners work at both ends of the spine. The problem is not the fertile diversity of subluxation hypotheses, but rather that the possible irrelevance of subluxation and adjustment is so infrequently addressed [e.g., [55,56]]. Many chiropractors (and others) have often been more disposed to ask where the subluxation is rather than whether subluxation correction is relevant or warranted.

The popularity of the subluxation construct is reflected in the variety of brand-name clinical techniques vended in the profession [e.g., [57-60]], many of which concern methods of subluxation detection and correction (see Table 2). We propose that the ubiquity and commercial success of these clinical procedures speak to the credence those doctors of chiropractic place in the various iterations of subluxation theories. Comparable claims for the clinical meaningfulness of subluxation may be found at the websites of several chiropractic colleges [36] and in the patient brochures distributed by major provincial, state and national membership societies of chiropractors in Canada and the United States (34). Many chiropractors bombard themselves and the public with subluxation rhetoric, but rarely hint at the investigational status of this cherished idea.

**Table 2:** Assertions about subluxation made by several brand-name technique organizations of chiropractic

...The mirror image adjustment resets the proprioceptive reflexes, inhibits the nocioceptive impulses and corrects the abnormal loading setting up the subluxation. In so doing, the reflex response of vasoconstriction to the viscera is removed and improved vascular tone to the smooth muscle, cardiac muscle and glands, results. The history of chiropractic success with patients experiencing such conditions as asthma, angina, visual disturbances, and other visceral conditions, is now clearly understood [57]
...D.N.F.T. utilizes a diagnostic system for subluxation analysis consisting of gentle challenging and a unique leg check. This testing allows the body itself to indicate the directions of misalignment of structures that are producing nerve interference. A gentle but directionally specific thumb impulse provides a long lasting correction to bony and soft tissue structures. D.N.F.T. is able to achieve structural corrections without torqueing, strong thrusts, and associated articular sounds that are often associated with traditional chiropractic... The goals of Directional Non-Force Technique are very much in line with the roots of traditional chiropractic: analyze and correct subluxations wherever they occur in the body, and allow the body to heal itself. Subluxations, as defined in Directional Non-Force Technique, are misalignments of tissue, osseous or soft, which result in nerve interference... [58].
...Minor displacements of the spinal bones, known as vertebral subluxations, can cause endangering stress to the spinal cord which acts as the main line of intelligence for the whole body. These displacements, or subluxations, are the cause of many of the unwanted health conditions that people suffer from every day. Although there have been many valuable techniques that have been developed in the chiropractic profession, the Gonstead System is considered a "gold standard" for chiropractic techniques because of its record of safety and effectiveness in correcting vertebral subluxation... [59].
...When the spinal column is in proper alignment, the "Brain Stem" can pass unimpinged through this foramen. But when one or both of the top two vertebrae become misaligned, the "Brain Stem" is impinged and normal nerve supply is reduced to parts of the body served by that nerve tract, hence sickness and disease... [60].

It has been our informal experience that subluxation is an unchallenged notion for many in the profession; Clum [39] concurs. Among the likely consequences of this unskeptical acceptance are evaluations and interventions that fail to address outcomes (in favor of focus on the presumed mediator: subluxation), excessive treatment (to correct something that may not be relevant: subluxation), unnecessary hazards (e.g., x-ray exposure in the quest for subluxation correction), and delay of appropriate care (through failure to diagnose and/or failure to seek alternative care). Subluxation, a construct that might be a source of guidance to chiropractors (were it to be rigorously investigated and validated), instead functions to distract us from the profession's prime directive: patient benefit.

### Subluxation in Marketing

The widespread use of unsubstantiated claims for subluxation and their adjustive correction in marketing to patients [e.g., [57-60]] and to prospective chiropractic students has been noted elsewhere [34,36]. Seaman [45] observes that:

...chiropractors [are] chastised as being "unscientific quacks"... Mostly, it has to do with claims that chiropractors make in marketing their services. Chiropractors are notorious for making treatment claims about chiropractic care that go well beyond the limits of our supportive data, whereas other professionals do not. Consequently, it is the chiropractor who looks like, and subsequently deserves to be called, an amateurish, unscientific huckster.

Some chiropractic suppliers are quite willing to jump on the unsubstantiated bandwagon of the subluxation, as the following promotion for nutritional products suggests:

The practice of Chiropractic is based upon the detection, correction and prevention of the Vertebral Subluxation Complex (VSC)...

*The goal of chiropractic care is to restore function to the damaged spine as quickly as possible to minimize the damaging effects of the VSC and the consequential degenerative changes... Current medical literature indicates that specific nutrients can also play an essential and integral role in the support of VSC... *[61].

Suffice it to say that the marketing assertions for the value of chiropractic care, frequently offered without acknowledgment of their non-validated status, are commonplace in the profession. The deleterious consequences attributed to subluxation and the clinical outcomes predicted for subluxation correction range from the dread of "killer subluxations" [62] to predictions of "optimal well being" [18] and attainment of maximum human potential. An advertisement that one chiropractor considers in poor taste may profess sacred truth for the next. Since substantiation of assertions may not be considered important to marketers, there are often no scientific boundaries to non-evidence-based chiropractic. Anything goes.

#### Subluxation as Legal & Political Strategy

The chiropractic subluxation began its legal relevance when the term was included in the wording of various statutes governing the practice of the chiropractic healing art. This trend was continued in the profession's quest for inclusion in the USA Medicare program more than 30 years ago. American chiropractors were chagrined for many years that payment for services in this federal program required radiographic "evidence" of subluxation, but did not compensate the chiropractor for the x-ray films; this stipulation has been eliminated. Many chiropractors now seek to secure their participation in Medicare (despite a skeptical medical community and the availability of manipulative services from non-chiropractor providers) by challenging the federal bureaucracy's interpretation of the Medicare statute.

In their recent "Memorandum of Points and Authorities in Support of Its Cross-motion for Summary Judgment" to the U.S. District Court for the District of Columbia in a suit against the U.S. Department of Health to establish chiropractors' exclusive right to reimbursement for "manipulation to correct a subluxation" in the Medicare program, attorneys for the ACA argue that:

*The ACA has presented substantial evidence that Congress did not intend that the services of medical doctors and osteopaths would overlap with the services of chiropractors. In fact, the ACA has clearly demonstrated the illogical paradox of the Secretary's interpretation, namely, that Congress would have had to intended that medical doctors and osteopaths were going to engage in a form of treatment that they believed to be cultist, in order to treat a condition that they did not believe existed, via a treatment method that they did not believe was possible. Surely this type of reasoning would have been absurd, and Congress could not have had that intention when it passed the amendments to the Social Security Act... *[63].

The irony here is extreme. Having established the legal meaningfulness of a hypothetical construct whose clinical relevance has yet (if ever) to be scientifically demonstrated, chiropractors now find themselves competing with physical therapists and others over the right to correct subluxations. The greatest absurdity of the situation appears to be missed by all parties concerned: subluxation is "real" because Congress has said so. Data seem irrelevant in this context. Monetary concerns clearly outweigh the issue of scientific validation, and the dogma of subluxation has now spread beyond the chiropractic profession.

#### Subluxation as Identity

Chiropractors since the Palmers have defined the profession by its focus on finding and adjusting subluxations. Intra-professional feuds have raged over just how exclusive this focus should be, but with few exceptions [e.g., [11,62,64,65]], allegiance is widely pledged to the traditional chiropractic lesion (e.g., Table 1). Clum [39] observes that for some chiropractors "the concept of vertebral subluxation is synonymous with chiropractic and its role has never been questioned." The subluxation is viewed by some chiropractors as a matter of "honor" [66]; anyone who questions the subluxation construct risks vilification as a heretic [66,67]. "Subluxation goes beyond metaphor; it is at the heart of chiropractic" [68]. The International Chiropractors' Association's (ICA's) president seeks a public relations campaign to make subluxation a "household word," and sees the ACC's paradigm as "a really good start" [69]. Edwards [25] insists that the American Chiropractic Association, the world's largest membership society of chiropractors, is no less committed to subluxation than is the Palmer-founded ICA. Gelardi [70] would define the chiropractic profession by its "mission"; his preferred mission is "to contribute to health through the correction of vertebral subluxation." Rome [37] argues that chiropractors' unique subluxation terminology is essential to the preservation of a unique identity. The endorsement of the ACC's statements on subluxation by national membership societies [28] constitutes additional affirmation of the sense among many chiropractic leaders of what a chiropractor is: a subluxation doctor.

Chiropractors' insistence upon defining the profession in terms of a hypothetical (and largely untested) construct is foolish at best: subluxation may or may not be a meaningful notion. This commitment also augurs against the conduct of clinical research to confirm or refute the utility of the subluxation construct, firstly because the presumption of validity undermines the motivation to investigate, and secondly because such research has the potential of undermining this proposed identity (i.e., subluxation doctor). The erosion of reimbursement for chiropractic services is also a possibility if subluxation research fails to measure up to expectations.

Ironically, there is an image of the chiropractor, which seems reasonably well-accepted by many members of the public and whose basis has already garnered some substantial research support [2,3]: the chiropractor as provider of manipulative/adjustive services. Whether the profession can loosen its self-imposed shackle to subluxation dogma is unclear.

#### Subluxation as Hypotheses

Chiropractors' reluctance to construe subluxation as hypothesis may derive in part from the limited consideration given to epistemology. Epistemology is that branch of philosophy, which deals with the nature of knowledge. Within the context of a clinical discipline such as chiropractic, epistemology addresses the means by which we may gain understanding about the nature of patients' problems, determine optimal methods of resolving or alleviating these problems, and appreciate the mechanisms by which successful interventions are accomplished. Chiropractors have traditionally offered a wide range of epistemological and reasoning strategies [7,71-80], including divine or spiritual inspiration, uncritical empiricism, uncritical rationalism (also referred to as "deductive science" [79]), truth by fiat (e.g., "the chiropractic principle": subluxation), and the critical rationalism and empiricism of the scientific method.

The confusion and incompatibility of these many epistemologies has arisen within a profession, which evolved outside of mainstream higher education and in its early years had little or no sophistication in the realm of scientific investigation [81,82]. Although scholarly and scientific sophistication has emerged in recent decades [83], it appears to be limited to a minority segment of the profession [e.g., [84]]. Inter-professional political pressures may offer a partial explanation for this [85]. Resistance to including chiropractic training within public universities may be more symptomatic than explanatory of the profession's scientific ennui, but the dearth of formal training programs for chiropractor-scientists at chiropractic colleges certainly suggests inadequate concern for the epistemological (i.e., scientific) bases for theories and practice in the profession.

For whatever the reasons, many in the chiropractic profession in the North American continent and in Australia and New Zealand remain committed to a dogmatic orientation to subluxation, its supposed health consequences and the putative benefits to be derived from subluxation-correction [17-19]. Although the percentage of chiropractors who adhere to dogmatism is not known, a 1994 sample of Canadian chiropractors was intriguing [86]. While 86% believed that chiropractors' methods should be validated, 74% disagreed that controlled trials are the best way to accomplish this. And though most (52%) disagreed that "The subluxation is the cause of many diseases," 68% agreed with the notion that "most diseases are caused by spinal malalignment" and most believed that subluxation was detectable by x-ray. Unfortunately, the survey methodology does not allow one to determine the tentative (hypothetical) vs. dogmatic quality of these beliefs.

The traditional chiropractic lesion is often seen as a "philosophical" truth or principle, something that must be defended rather than investigated [87]. This unfortunate pitting of "chiropractic principle" [67] against research scrutiny is often couched in terms of a conflict between philosophy and science:

*...It is my contention that a battle between philosophy and science does not and cannot exist within the chiropractic profession or any other discipline. I contend that the real battle is between the great majority of chiropractors who unknowingly allow dogmatism to guide the practice of chiropractic and the extremely rare variety of chiropractor who's practice of chiropractic is guided by philosophy and science *[45].

There is nothing inherently dogmatic or anti-scientific in the notion that an articular lesion may have health consequences, or that correction of joint dysfunction may relieve symptoms and/or improve health. Neither does our current inability to predict the effects (if any) of subluxation [88] and/or the benefits of subluxation-correction relegate this hypothetical construct to the dustbin of clinical theories. Indeed, it would be just as inappropriate to dispose of this largely untested theory without data as it is to proclaim its meaningfulness without adequate evidence. On the other hand, as Carl Sagan suggested, extraordinary claims will require extraordinary evidence. With respect to the supposed mechanisms of adjusting, Haldeman [23] reminds us that "What must be avoided... is the unreasonable extrapolation of current knowledge into speculation and presentation of theory as fact." Given the current deficiency of empirical data, the only sound scientific-epistemological position that we can conceive of is to acknowledge our ignorance: we don't know if subluxation is clinically meaningful or not. We suggest that this is a requisite first step toward greater wisdom concerning subluxation.

#### A Simple Alternative

Speculations and tentative assertions are the stuff from which rigorous science emerges [71]. Indeed, there are those rare scientists whose enduring contributions have derived as much or more from what they theorized than from what they actually tested experimentally (e.g., Isaac Newton and the motions of the planets; Albert Einstein and relativity; Linus Pauling and the role of the hemoglobin molecule in sickle-cell anemia). Hypothetical constructs such as the chiropractic lesion, emotional stress and the neurotic syndromes may or may not have important implications for human biology, but it is entirely appropriate to offer such ideas as tentative assertions.

We could, as C.O. Watkins, D.C. urged decades ago, resolve to be bold in what we hypothesize but cautious and humble in what we claim. In discussing subluxation, all chiropractors should learn to use language that denotes the tentative character of many of our beliefs (hypotheses). Those chiropractors who suspect that subluxation has significant health implications could resolve to investigate scientifically (e.g., through meticulous case reporting), or at least to financially support rigorous investigations, of the meaningfulness of subluxation and its correction. The leaders of our colleges, membership societies and agencies could qualify their statements about subluxation by admitting up front that subluxation is hypothesis(es), not an experimentally demonstrated reality. Those who speak for the profession and who operate in the political, legal and legislative arenas could advance the cultural authority of the profession by becoming credible, balanced, evidence-based sources of information about the chiropractic art. The chiropractic rank-and-file could be encouraged to recognize that responding to charges of quackery with unsubstantiated claims for subluxation and for the outcomes of chiropractic care is self-defeating. Marketers could eliminate the spizzerinctum and hype in their advertisements and concentrate on those aspects of chiropractic for which good data already exist. Speculations could be identified as such, so as not to violate the public's trust and enfeeble the profession's best efforts to progress.

How can such profound change in the profession come about? A century of criticisms by political medicine, many of them not unlike those we offer, has only hardened many chiropractors' attitudes [85]. However, the purpose here is not to contain and eliminate the chiropractic profession, but rather to challenge dogmatic adherence to a hypothetical construct and to help to remedy the many problems that dogmatism has cost the profession. We believe that chiropractic should proceed as a first-class clinical science and art, a profession whose members appreciate and acknowledge what is known and what is not, provide patients with the best care possible given current knowledge, and resolve to extend the borders of scientific understanding in the interest of the public we serve.

The metamorphosis we seek begins with the individual chiropractor who is willing to challenge tradition and peers in the interest of greater integrity for the profession and greater benefit for patients. There is a silent minority who recognize the inappropriateness of the prevailing consensus of dogma concerning subluxation. We recommend that individuals and small groups speak out, educate peers about the distinction between subluxation as hypothesis versus subluxation as dogma, and assert their dissatisfaction with unsubstantiated claims made for the traditional chiropractic lesion. "Silence is not golden: it's consent" [89].

We ask that those who guide the profession and who understand the dilemma that subluxation dogma causes the profession, lead by word and example. Whether one is college faculty or administrator, association official or appointee to a licensing authority, a willingness to reframe subluxation as something tentative rather than something certain is essential. Silence can only serve to sustain our century-long, epistemological misunderstanding of the subluxation construct and corrupt the fullest expression of a worthy future.

## Summary

Hypothetical constructs involve tentative assertions about physical reality. They serve as essential tools in the development of science, and permit the empirical testing of the non-obvious. However, when the speculative nature of an hypothesis or hypothetical construct is not made obvious, an otherwise acceptable proposition becomes a dogmatic claim. Such is the history of subluxation in chiropractic.

This brief review of the role of subluxation dogma in clinical practice, in marketing, in the legal and political arenas, as a basis for professional identity, and in the rhetoric of leading chiropractic organizations and agencies, is not a statement about subluxation's validity or lack thereof. Only focused clinical research will enable us to determine whether the traditional chiropractic lesion merits clinicians' attention. We don't know whether subluxation is meaningful or not.

The dogma of subluxation is perhaps the greatest single barrier to professional development for chiropractors. It skews the practice of the art in directions that bring ridicule from the scientific community and uncertainty among the public. Failure to challenge subluxation dogma perpetuates a marketing tradition that inevitably prompts charges of quackery. Subluxation dogma leads to legal and political strategies that may amount to a house of cards and warp the profession's sense of self and of mission. Commitment to this dogma undermines the motivation for scientific investigation of subluxation as hypothesis, and so perpetuates the cycle.

The simple expedient of amending dogmatic assertions to note their tentative, hypothetical character could do much to improve the image of the profession, to re-orient it to the challenge of testing its cherished hypotheses and to establishing the cultural authority of chiropractors in our unique realm of health care. The task of reorienting the profession to a credible science and art belongs to all who understand the scourge of dogma, and who seek a brighter future for the chiropractic profession and its patients.

## Authors' contributions

All authors contributed to the writing and re-writing of this paper.
